# Audiometric, Perceptual and Subjective Cognitive Assessment of Hearing Loss in Adults with Hearing-Related Complaints

**DOI:** 10.3390/audiolres16050150

**Published:** 2026-10-01

**Authors:** Marta Álvarez-Cendrero, Andrea Rodríguez Rodero, María A. Callejón-Leblic

**Affiliations:** 1 Department of Otolaryngology, Head and Neck Surgery, De La Merced University Hospital, 41640 Osuna, Spain; andrearodrod@gmail.com; 2 Department of Otolaryngology, Head and Neck Surgery, Virgen Macarena University Hospital, 41009 Seville, Spain; mcallejon@us.es

**Keywords:** hearing loss, pure-tone audiometry, SSQ12, cognitive screening, subjective cognitive complaints, presbycusis, noise exposure, patient-reported outcomes

## Abstract

**Background:** Pure-tone audiometry (PTA) does not fully capture functional hearing difficulties. This study examined relationships between audiometric thresholds, self-reported hearing quality (SSQ12), and cognitive screening in patients with auditory complaints, as well as the association of other factors such as age and noise exposure with these measures. **Methods:** Data from 178 patients complaining about subjective hearing loss (mean age 55 ± 17.4 years; 43.3% male), including PTA and SSQ12, were analyzed. A subgroup of 60 patients aged ≥ 60 years also completed the Subjective Cognitive Decline Questionnaire (MiCog/SuCog). Associations were assessed via bivariate correlation and multiple linear regression analyses. **Results:** Age was independently associated with higher PTA4 thresholds in both ears (right: B = 0.60 dB HL per year, 95% CI 0.47–0.73; left: B = 0.61 dB HL per year, 95% CI 0.48–0.74; *p* < 0.001). SSQ12 scores correlated with PTA (right ear ρ = −0.532) and age (ρ = −0.283; both *p* < 0.001) and were lower in noise-exposed participants after adjusting for PTA, age, sex and tinnitus (B = −1.005, 95% CI −1.565 to −0.446, *p* < 0.001). In the cognitive subgroup, right-ear PTA showed an unadjusted correlation with MiCog (ρ = 0.332, *p* = 0.010); in the adjusted model, neither PTA nor any other predictor was independently associated with MiCog (PTA: B = 0.095, 95% CI −0.075 to 0.264, *p* = 0.266). No significant association was found between MiCog and the SSQ12 total score (Pearson r = −0.166, *p* = 0.204; Spearman ρ = −0.241, *p* = 0.064). **Conclusions:** Audiometric thresholds, self-reported hearing quality, and cognitive screening capture partially distinct, interrelated dimensions of auditory function. Self-reported measures revealed noise-related difficulties undetected by audiometry. These findings support incorporating patient- and informant-reported outcomes alongside audiometry to support a more comprehensive, patient-centered approach to hearing and cognitive care.

## 1. Introduction

Population aging has become one of the main challenges facing healthcare systems worldwide, with important clinical, social, and economic consequences [1]. As life expectancy continues to increase, greater emphasis has been placed on promoting healthy aging and preventing cognitive decline through the identification and management of modifiable risk factors [2]. Among these, hearing loss has emerged as the most important potentially modifiable risk factor for cognitive impairment and dementia [3,4,5].

Age-related hearing loss affects approximately 40% of adults aged 60–69 years, and its prevalence approaches 90% in individuals older than 80 years [6,7]. Moreover, the risk of dementia increases progressively with hearing loss severity, approximately doubling in individuals with mild hearing impairment, tripling in those with moderate hearing loss, and increasing up to fivefold in severe cases [8,9]. These findings highlight the importance of early identification of auditory dysfunction and timely rehabilitation to reduce the individual and societal burden associated with cognitive decline [10]. Thus, individuals with hearing difficulties may be considered a population at risk not only because of the potential progression of hearing impairment, but also because hearing loss may have important functional, cognitive, and social consequences, including difficulties in communication, reduced quality of life, and an increased risk of cognitive decline [3,5,10].

Despite this need, the early diagnosis of hearing impairment remains challenging [11]. A proportion of patients report persistent hearing difficulties—particularly impaired speech understanding in noisy environments—despite having normal pure-tone audiometric thresholds [12]. This clinical presentation, commonly referred to as hidden hearing loss, has been associated with cochlear synaptopathy, characterized by damage to the synapses between inner hair cells and auditory nerve fibers that preserves hearing thresholds while impairing suprathreshold auditory processing [13,14,15]. Beyond the cochlear level, these deficits may also reflect impaired central auditory processing, whereby the neural pathways responsible for encoding, analyzing, and integrating auditory information beyond the cochlea fail to support complex listening tasks such as speech-in-noise perception, temporal processing, and binaural integration, even when peripheral hearing thresholds remain normal [16]. Because conventional pure-tone audiometry is insensitive to these deficits, affected individuals may remain undiagnosed or experience delays in receiving appropriate management [12,16]. Consequently, there is growing interest in complementary assessment strategies capable of identifying auditory dysfunction beyond the conventional audiogram.

Patient-reported outcome measures have therefore gained increasing relevance in audiological research and clinical practice by providing information that cannot be captured through objective hearing tests alone [17]. Hearing-related quality-of-life (HRQoL) questionnaires allow the systematic evaluation of perceived hearing difficulties in everyday listening situations, particularly in noisy and acoustically complex environments that are difficult to reproduce under standard clinical conditions [17]. These subjective measures complement audiological testing by reflecting the functional impact of hearing impairment on daily communication [18,19].

This perspective is particularly relevant for individuals who experience hearing difficulties despite relatively preserved conventional audiometric thresholds, as subjective measures may capture functional and social consequences that are not fully reflected by the audiogram.

The SSQ12 in particular samples this functional impact across three complementary domains—speech hearing, spatial hearing, and qualities of hearing—so that, unlike a single global disability score, it can indicate whether a patient’s difficulty lies primarily in following conversation, localizing sound sources, or perceiving sound clarity and naturalness, information that is not recoverable from the pure-tone audiogram and that may help tailor counselling and rehabilitation to the specific listening situations a patient finds most disabling [20,21].

Because hearing loss and cognitive decline are closely interconnected, the incorporation of cognitive screening tools into audiological assessment may provide additional clinical value. Brief instruments such as the Spanish version of the Subjective Cognitive Decline Questionnaire (SCD-Q) facilitate the early identification of self-perceived cognitive changes through its self-report form (MiCog, “MyCog” in the original validation study), while the informant-report form (SuCog, “TheirCog” in the original study) offers a complementary perspective on cognitive changes in daily life, as completed by an informant [22]. Together with HRQoL questionnaires, these tools support a more comprehensive patient-centered assessment that extends beyond pure-tone audiometry, in accordance with current international recommendations advocating the evaluation of communication ability, functional status, quality of life, and cognition as part of hearing healthcare [23,24].

The present study included adults with hearing-related complaints across a broad age range, including individuals who reported subjective hearing difficulties despite having normal conventional audiograms. Audiometric and perceptual measures were assessed across the entire sample, while subjective cognitive performance was evaluated in participants aged 60 years and older [25]. Environmental noise exposure was examined as a potential factor associated with auditory performance among participants who reported such exposure. This approach allowed us to characterize auditory and perceptual features in a heterogeneous adult population, with relevance to individuals whose auditory complaints may not be fully captured by conventional audiometry and to the potential functional, cognitive, and social consequences associated with hearing impairment.

Accordingly, the aims of this study were: (i) to investigate the relationship between audiometric thresholds, perceived hearing quality measured as SSQ12 scores, and cognitive screening outcomes in individuals presenting with auditory complaints; (ii) to assess the association of age and environmental noise exposure with auditory performance; (iii) to identify potential indicators of early auditory dysfunction in individuals with normal conventional audiometry; (iv) to explore the relationship between HRQoL and subjective cognitive complaints; and (v) to provide the scientific community with an open dataset to promote further use and analysis.

## 2. Materials and Methods

### 2.1. Study Design and Participants

A retrospective analytical study was conducted based on the review of medical records of patients attending the Department of Otorhinolaryngology of a Level II University Hospital who underwent pure-tone audiometry (PTA) between 1 January 2025, and 30 June 2025. A non-random consecutive sampling approach was used. A total of 250 medical records were reviewed, of which 178 patients met the eligibility criteria and were included in the study. Patients older than 18 years with subjective hearing loss and self-reported poor speech intelligibility in noise were included, if PTA results and a completed SSQ12 questionnaire were available in their medical records. Of the 250 records reviewed, 72 patients were excluded: 69 because the required audiometric test and/or questionnaires were not documented in their medical records, and 3 because of incapacitating neuropsychiatric comorbidities that could interfere with the study assessment (one patient with schizophrenia and two with dementia). Milder or unrelated neurological diagnoses (e.g., migraine, cervical disc herniation, or a previous stroke without cognitive sequelae) did not meet this exclusion threshold and were retained as descriptive clinical variables (Table 1), given their potential relevance to the interpretation of vestibular, auditory, or cognitive findings. Similarly, a small number of patients had anxiety or depressive disorders under adequate clinical control; given the limited number of affected participants, these conditions were likewise recorded only as descriptive clinical variables, without dedicated subgroup analyses.

In addition, a cognitive subgroup was analyzed. This subgroup comprised patients aged 60 years or older who had a completed MyCog questionnaire available in their medical records. Thus, not all participants aged 60 years or older were necessarily included in the cognitive subgroup, as the availability of a completed MyCog questionnaire in the medical record was an additional inclusion criterion. A questionnaire was considered completed when item-level responses were recorded; participants without a completed MiCog or SuCog questionnaire were coded as missing and were excluded from all analyses involving that questionnaire. A cut-off of 60 years was selected because signs and symptoms associated with physiological ageing become increasingly evident from this decade onward [25]. The SuCog questionnaire was completed by a first-degree relative or, when unavailable, by the patient’s primary caregiver.

The study was conducted in accordance with the principles of the Declaration of Helsinki and was approved by the Ethics Committee (SICEIA-2026-001375). All data were anonymized during database construction, prior to processing and analysis, to ensure the confidentiality of the participant’s information.

### 2.2. Data and Variables Collected

#### 2.2.1. Demographic and Clinical Variables

Demographic characterization included age and gender (male/female). Clinical history variables were recorded as categorical (yes/no) data and comprised exposure to ototoxic agents—assessed both through active questioning of the patient and review of prior clinical records—environmental exposure to intense noise, and self-reported diagnoses of neurological, mental health, otologic, auditory, and vestibular disorders, as well as use of hearing assistive devices. Audiological clinical variables included hearing loss type, classified as a nominal variable (sensorineural, conductive, or mixed), with classification performed independently for each ear, and presence of tinnitus (yes/no). Hearing loss type was classified using air-conduction (AC) and bone-conduction (BC) thresholds averaged over 500, 1000, 2000 and 4000 Hz, and the mean air–bone gap (ABG) over the same frequencies, as follows: normal hearing, AC PTA4 < 20 dB HL; conductive hearing loss, AC PTA4 ≥ 20 dB HL with BC PTA4 < 20 dB HL and mean ABG ≥ 15 dB; mixed hearing loss, AC PTA4 ≥ 20 dB HL with BC PTA4 ≥ 20 dB HL and mean ABG ≥ 15 dB; sensorineural hearing loss, AC PTA4 ≥ 20 dB HL with mean ABG < 15 dB; cophosis, absence of response at the maximum output of the audiometer at all tested frequencies. The degree of hearing loss was graded according to the World Health Organization (WHO) criteria [6,26]. Supplementary Table S1 summarizes and details the variables collected for the study.

#### 2.2.2. Measures of Performance

Audiometric thresholds

For the purposes of this study, hearing thresholds were summarized using several pure-tone average (PTA) metrics. PTA4 was defined as the average air-conduction threshold at 500, 1000, 2000, and 4000 Hz. In addition, a total PTA, calculated as the mean threshold across all tested frequencies (250–8000 Hz), was included to provide a more comprehensive characterization of the audiometric profile. Normal hearing was defined as PTA4 < 20 dB HL, in accordance with the World Health Organization classification [24].

Audiometric assessments were performed by qualified personnel in a soundproof booth using a MADSEN Astera^2^ audiometer (Natus Medical Denmark ApS, Taastrup, Denmark). Pure-tone audiometry across the conventional frequency range (250 Hz–8 kHz) was performed using TDH-49 headphones (Telephonics Corporation, Farmingdale, NY, USA; supplied by Natus Medical Denmark ApS, Taastrup, Denmark). Bone-conduction thresholds were assessed at 500, 1 K, 2 K and 4 KHz. When required, masking was applied using the plateau method [27]. The mean air-bone gap was defined as the average difference between air-conduction and bone-conduction thresholds at 500, 1000, 2000, and 4000 Hz, calculated for ears with conductive or mixed hearing loss. All equipment undergoes regular inspection to ensure proper functioning and calibration in accordance with current standards, including IEC 60645-1 for the audiometer (International Electrotechnical Commission, 2017), ISO 8253-1 for the soundproof booth (International Organization for Standardization, 2010), and ISO 389-1 (International Organization for Standardization, 2017) for the headphones [28,29,30].

Self-administered Hearing Questionnaire: SSQ12

Self-perceived hearing disability was assessed using the Spanish version of the 12-item Speech, Spatial and Qualities of Hearing Scale (Sp-SSQ12), a short form of the original 49-item SSQ [31] developed by Noble et al. [32] and validated in Spanish-speaking populations by Cañete et al. [33].The questionnaire comprises 12 items across three domains: Speech hearing (5 items), Spatial hearing (3 items), and Qualities of hearing (4 items). Each item is rated on a 10-point scale from 0 (“not at all”/”cannot at all”) to 10 (“perfectly”), and an overall score is calculated as the mean of all items, with subscale scores obtained as the mean of items within each domain; lower scores indicate greater self-perceived hearing difficulty. A cutoff score of ≤8.5 has been proposed to identify clinically significant self-reported hearing disability (sensitivity 80%, specificity 75.6%) [32,33,34,35].

Subjective cognitive status: SCDQ

Subjective cognitive status was assessed using the Subjective Cognitive Decline Questionnaire (SCD-Q), developed and validated by Rami et al. [22]. The SCD-Q consists of two parallel 24-item forms addressing perceived changes in memory, language, and executive function over the preceding two years: MyCog (“MiCog”), completed by the patient, and TheirCog (“SuCog”), completed by an informant or caregiver. Each item is scored dichotomously (0 = no perceived decline, 1 = perceived decline), yielding a total score of 0–24, with higher scores reflecting greater subjective cognitive decline. An optimal SuCog cutoff of ≥7/24 has been established to discriminate between individuals with and without objective cognitive impairment (sensitivity 85%, specificity 80%) [22].

### 2.3. Statistical Analysis

Statistical analyses were performed using Jamovi (Version 2.7.34.0; The Jamovi Project, 2024), built on R and Python (Version 3.11.15) (statsmodels, SciPy) for the Steiger test, bootstrap and robust-standard-error analyses. Descriptive statistics (means, standard deviations, medians, ranges, and 95% confidence intervals) were calculated for all continuous variables, and the normality of their distributions was assessed using the Shapiro–Wilk test. Associations between continuous variables were examined using Pearson’s correlation coefficient (r) when normality assumptions were met, and Spearman’s rank correlation coefficient (ρ) as a robust, non-parametric alternative when this assumption was violated. Associations were modeled using multiple linear regression models, with the dependent variable varying according to the specific research question (i.e., age, bilateral PTA, and MiCog score were each modeled as outcomes in separate analyses), applying both stepwise variable selection and standard (simultaneous) entry of all candidate predictors; model fit was evaluated using R, R^2^, and Akaike Information Criterion (AIC: with lower values indicating better model parsimony), multicollinearity was assessed via the variance inflation factor (VIF) and tolerance statistics, and model assumptions were checked using the Durbin-Watson test for residual autocorrelation and the Shapiro–Wilk test for residual normality. When residuals deviated from normality, heteroscedasticity-consistent (HC3) standard errors were computed as a sensitivity analysis. For all analyses, statistical significance was set at *p* < 0.05.

For the cognitive subgroup (n = 60), because the MiCog total score is a bounded count variable (sum of 24 items, range 0–24) with a skewed, zero-inflated distribution, negative binomial regression was used as a sensitivity analysis alongside linear regression; given the limited case-to-predictor ratio in this subgroup, zero-order correlations between MiCog and SSQ12 total scores were conducted. Specific item- and frequency associations were included as well only as part of exploratory analyses. The difference between two dependent, overlapping Spearman correlations (MiCog with the 250 Hz–2 kHz average vs. MiCog with the 4–8 kHz average) was tested with Steiger’s Z test, entering the Spearman coefficients in place of Pearson coefficients. Because this test was derived for Pearson correlations, it was treated as an approximation and complemented by a percentile bootstrap 95% confidence interval (5000 resamples) for the difference between the two coefficients.

Analyses were classified a priori into primary, aim-linked analyses and exploratory analyses (item-level SSQ12/MiCog associations, frequency-specific bivariate correlations, and post hoc pairwise comparisons); a Benjamini–Hochberg false-discovery-rate correction was applied within the family of primary analyses, with exploratory analyses reported descriptively and not corrected for multiplicity given the high intercorrelation among comparisons. Effect sizes with 95% confidence intervals are reported for primary analyses. Please see Supplementary Table S2 for a complete list with a description of primary and exploratory analysis.

Missing data were handled by complete-case (listwise) analysis rather than imputation, given the low overall proportion of missing values (≤3.4% for any variable entering a given model); the exact number of cases excluded per analysis is reported alongside each result.

## 3. Results

### 3.1. Study Population and Outcome Variables

A total of 178 subjects were included in the study, with a mean age of 55 ± 17.4 years (range 18–90). Of these, 77 (43.3%) were male and 101 (56.7%) were female. Environmental noise exposure was reported by 70 subjects (39.3%) and exposure to ototoxic agents by 4 (2.2%). Otologic findings were normal in the right ear in 161 subjects (90.4%) and in the left ear in 158 subjects (88.7%). Chronic middle ear disease was present in 22 subjects (12.3%) in the right ear and in 26 subjects (14.6%) in the left ear. A history of otologic surgery was reported by only 1 subject (0.6%) for the right ear, and by 5 subjects (2.8%) for the left ear.

Regarding hearing loss type, 132 subjects (74.2%) presented hearing loss in the right ear, of whom 8 (6%) had a conductive component and 13 (9.8%) a mixed component; hearing loss in the left ear was present in 136 subjects (76.4%), of whom 10 (7.3%) had a conductive component, 15 (11%) a mixed component, and 2 (1.5%) cophosis. Tinnitus was reported by 88 patients (49.4%) of the sample, and 25 subjects (14.1%) presented associated vestibular symptoms. With respect to hearing aid fitting, only 2 patients (1.1%) of the sample were hearing aid users.

The mean PTA4 in the right ear was 33 ± 18.6 dB (range 1.3–85), and the mean total PTA in the right ear was 33.9 ± 17.9 dB (range 3.3–86.7), with a mean air-bone gap of 24.2 ± 8.4 dB (range 15–45) among conductive hearing losses. In the left ear, the mean PTA4 was 33.7 ± 18 dB (range −1.25–97.5), and the mean total PTA was 34.4 ± 17.9 dB (range 0.83–99.2), with a mean air-bone gap of 26.1 ± 9.3 dB (range 15–45) among conductive hearing losses. Table 1 shows main descriptive statistics calculated for the total sample of patients (N = 178) of the study. In addition, the distribution of audiometric thresholds across frequencies for both ears is shown in Figure 1.

The mean score on the SSQ12 questionnaire, which assesses self-perceived hearing-related quality of life across speech understanding, spatial hearing, and sound quality domains, was 5.64 ± 2.3 (range 0.8–10), with higher scores indicating better perceived auditory function and lower scores reflecting greater hearing-related difficulty. A cut-off value of ≤8.5 has been proposed as an indicator of self-reported hearing difficulty in SSQ-12 [33].

Of the 178 participants included in the study, 85 were aged ≥ 60 years. Among these, 60 had completed MiCog questionnaire available in their medical records and were therefore included in the cognitive subgroup. The mean score on the MiCog questionnaire, which screens for self-reported cognitive complaints, was 7.9 ± 6.6 (range 0–23), with higher scores indicating a greater number or severity of cognitive complaints. Among the 60 participants with a completed MiCog questionnaire, 38 (63.3%) also had a completed SuCog questionnaire. The mean score on the SuCog questionnaire, completed by an accompanying informant based on their perception of the patient’s cognitive-listening difficulties, was 10.4 ± 5.3 (range 0–24), with higher scores reflecting greater informant-reported cognitive-related difficulty. Table 2 shows the comparison of demographic and clinical characteristics between the two subgroups, both with and without MyCog documented. Statistical differences were only found in age (*p* < 0.001) and PTA in both right (*p* < 0.001) and left ears (0.019) for the two subgroups.

### 3.2. Interaural Relationship and Age-Related Audiometric Decline

PTA4 and total PTA were highly correlated within each ear, indicating substantial overlap between both measures (right ear: Pearson’s r = 0.989, Spearman’s ρ = 0.988; left ear: Pearson’s r = 0.987, Spearman’s ρ = 0.984; all *p* < 0.001). Correlations between the right and left ears were moderate-to-strong: interaural PTA4 correlation was ρ = 0.626 (Pearson’s r = 0.637; n = 175; *p* < 0.001), and interaural correlations of corresponding single frequencies ranged from ρ = 0.543 at 250 Hz to ρ = 0.764 at 8 kHz (all *p* < 0.001). The mean right–left PTA4 difference was −0.7 ± 15.6 dB (paired *t*-test *p* = 0.574; Wilcoxon signed-rank *p* = 0.560), indicating no systematic difference between ears at group level; however, 44 participants (25.1%) had an interaural PTA4 difference ≥ 15 dB.

Age was moderately correlated with individual hearing thresholds in both ears (right ear: Pearson’s r = 0.515, Spearman’s ρ = 0.560; left ear: Pearson’s r = 0.562, Spearman’s ρ = 0.574; all *p* < 0.001). This relationship was evident across all frequencies, with correlation strength increasing progressively from low to high frequencies (Pearson’s r = 0.294–0.304 at 250 Hz to 0.658–0.691 at 8000 Hz; all *p* < 0.001). Within each ear, the strongest correlations were observed between adjacent frequencies (e.g., r = 0.932 between 250 and 500 Hz in the right ear; r = 0.936 in the left ear), with progressively weaker correlations as frequency separation increased.

To examine whether age was associated with hearing thresholds independently of clinical covariates, separate multiple linear regression models were fitted for each ear with PTA4 as the dependent variable and age as the predictor of interest, adjusting for noise exposure, tinnitus, ear-specific otologic diagnosis and vestibular pathology. In the right ear (N = 177; R^2^ = 0.395, adjusted R^2^ = 0.366; F (8.168) = 13.73, *p* < 0.001), age was independently associated with higher PTA4 (B = 0.599 dB HL per year, 95% CI 0.469–0.729, SE = 0.066, t = 9.08, *p* < 0.001; standardized β = 0.56). The same was observed in the left ear (N = 174; R^2^ = 0.421, adjusted R^2^ = 0.382; F (11.162) = 10.71, *p* < 0.001; B = 0.609 dB HL per year, 95% CI 0.483–0.735, SE = 0.064, t = 9.56, *p* < 0.001; β = 0.59). Thus, after adjustment, each additional decade of age was associated with approximately 6 dB HL higher PTA4 in each ear (Figure 2). Among the covariates, otosclerosis (right: B = 26.9, 95% CI 13.5–40.3, *p* < 0.001; left: B = 23.4, 95% CI 11.6–35.2, *p* < 0.001) and serous otitis media (right: B = 26.5, 95% CI 14.1–38.9, *p* < 0.001; left: B = 26.1, 95% CI 9.7–42.5, *p* = 0.002) were associated with higher PTA4 relative to normal otologic findings, whereas noise exposure (right: B = 4.23, *p* = 0.069; left: B = 4.23, *p* = 0.063), tinnitus, vestibular pathology and the remaining otologic categories were not significant. Collinearity diagnostics revealed no relevant issues (VIF 1.02–1.09). Durbin–Watson statistics were 2.04 (right) and 1.63 (left). Because residuals deviated from normality (Shapiro–Wilk *p* < 0.001 in both models), HC3 robust standard errors were computed; the association with age was unchanged (right: 95% CI 0.468–0.729; left: 95% CI 0.489–0.729; both *p* < 0.001).

### 3.3. Audiometric Profile by Hearing Status

Figure 3 shows right-ear thresholds by frequency in ears with normal hearing (n = 46) and with hearing loss (n = 132). Ears with normal hearing had a flat profile, with median thresholds of 10–15 dB HL and narrow interquartile ranges at all frequencies. In ears with hearing loss, median thresholds rose progressively with frequency, from 30 dB HL at 250 Hz–1 kHz to 35 dB HL at 2 kHz, 50 dB HL at 4 kHz and 55 dB HL at 8 kHz. This is a sloping, predominantly high-frequency configuration. Consequently, the difference in medians between groups widened from 15 dB at 250–500 Hz to 40 dB at 4 kHz and 42.5 dB at 8 kHz, and the distributions overlapped at low frequencies but were largely separated from 2 kHz upwards. As hearing status was defined by PTA4, which includes the 500–4000 Hz thresholds, this figure is purely descriptive and was not tested statistically.

### 3.4. SSQ12 Scores: Clinical Correlates and Multivariable Predictors

Across the combined sample, SSQ12 scores correlated negatively with age (ρ = −0.283, *p* < 0.001) and with bilateral PTA (right ear: ρ = −0.532; left ear: ρ = −0.484; both *p* < 0.001), indicating that self-reported hearing-related quality of life was poorer among older participants and those with greater audiometric loss. Given the number of clinical variables of potential relevance (hearing status, noise exposure, ototoxic exposure, tinnitus, type of hearing loss, and otologic diagnosis), their association with SSQ12 scores was assessed jointly through multivariable regression rather than through separate pairwise comparisons, as detailed below.

The association between noise exposure and SSQ12 was further examined with noise-exposed participants considered as a distinct group. Out of the 178 participants, 70 (39.3%) reported a history of environmental noise exposure. Mean PTA4 did not differ significantly between noise-exposed and non-exposed participants in either ear (right ear: 35.0 ± 17.1 vs. 31.7 ± 19.4 dB HL, t = 1.22, *p* = 0.225; left ear: 35.5 ± 18.3 vs. 32.6 ± 17.8 dB HL, t = 1.02, *p* = 0.311; total PTA showed the same pattern, *p* = 0.164 and *p* = 0.353, respectively), and age was also comparable between groups (55.5 ± 17.2 vs. 54.7 ± 17.6 years, *p* = 0.783). However, the distribution of audiological diagnoses differed significantly according to noise-exposure history (χ^2^(5) = 12.15, *p* = 0.033), with acoustic trauma being more than four times more frequent among noise-exposed participants (9/70, 12.9%) than among non-exposed participants (3/108, 2.8%). Taken together, noise-exposed patients were not distinguishable from non-exposed patients on the basis of pure-tone thresholds or age but showed a distinct audiological diagnostic profile (more acoustic trauma); the independent association between noise exposure and self-reported hearing-related quality of life is addressed in the multivariable model below.

To determine whether this association was independent of audiometric severity, age, sex, and tinnitus, a multiple linear regression was fitted with the SSQ12 total score as the dependent variable and noise-exposure history as the main predictor, controlling for bilateral average PTA4, age, sex, and tinnitus presence (N = 177). Noise exposure remained an independent, statistically significant predictor of lower (poorer) SSQ12 scores (B = −1.005, 95% CI −1.565 to −0.446, *p* < 0.001), as did bilateral average PTA4 (B = −0.081, 95% CI −0.101 to −0.061, *p* < 0.001) and sex (female vs. male; B = −0.671, 95% CI −1.223 to −0.118, *p* = 0.018); age (B = 0.002, 95% CI −0.017 to 0.022, *p* = 0.822) and tinnitus presence (B = −0.008, 95% CI −0.554 to 0.538, *p* = 0.978) were not significant independent predictors. Model R^2^ = 0.394; all variance inflation factors were low (1.03–1.56), indicating no relevant multicollinearity. This model, presented as the primary analysis of the noise exposure–SSQ12 association, indicates that this association is not attributable to differences in hearing-threshold severity, age, sex, or tinnitus between exposed and non-exposed participants (Table 3, Figure 4).

### 3.5. Cognitive Screening: Correlations with Audiometric and SSQ12 Measures

A subgroup of 60 participants was analyzed, comprising individuals aged ≥ 60 years who had completed the MiCog assessment. The subgroup had a mean age of 72.2 ± 8.1 years (range, 60–90 years) and a mean right-ear PTA of 44.3 ± 15.8 dB HL (range, 18.8–83.8 dB HL) (see Table 2).

Regarding demographic and audiometric characteristics, no significant correlation was observed between MiCog scores and age (ρ = 0.044, *p* = 0.736). In contrast, higher PTA was significantly associated with higher MiCog scores (ρ = 0.332, *p* = 0.010). This association was driven specifically by thresholds at 250 Hz–2 kHz (r = 0.26–0.32, *p* ≤ 0.046), whereas no significant associations were observed at 4–8 kHz (r ≤ 0.08, *p* ≥ 0.56), thus possibly suggesting a relationship between low-to-mid-frequency auditory sensitivity and cognitive complaints in this sample (Figure 5). These frequency-specific findings should be considered exploratory.

A comparison of these two dependent, overlapping correlations using Steiger’s Z test (applied to Spearman coefficients; n = 58 participants with complete thresholds; ρ between MiCog and the 250 Hz–2 kHz average = 0.354; ρ between MiCog and the 4–8 kHz average = 0.066; ρ between the two frequency averages = 0.407) yielded Z = 2.02, *p* = 0.043. The bootstrap estimate of the difference between the two coefficients was Δρ = 0.29 (95% CI 0.01–0.55). Given that Steiger’s test is an approximation for Spearman coefficients, that the *p*-value is close to the significance threshold and that this comparison is exploratory, this result is regarded as hypothesis-generating only.

The PTA4–MiCog association also depended on the ear analyzed. It was not significant for the left ear (ρ = 0.109, *p* = 0.409) or for the better ear (ρ = 0.207, *p* = 0.112) and was weaker for the bilateral average (ρ = 0.283, *p* = 0.029) and the worse ear (ρ = 0.276, *p* = 0.033) than for the right ear. Given the only moderate interaural correlation reported in Section 3.2, right-ear results cannot be assumed to represent both ears, and this unadjusted association should not be regarded as robust.

Self-reported noise exposure was not significantly associated with MiCog scores. Participants with and without a history of noise exposure showed comparable scores (t (58) = −0.288, *p* = 0.774; Mann–Whitney U = 417, *p* = 0.988), providing no evidence of an association between previous noise exposure and subjective cognitive complaints in this subgroup.

As the principal multivariable analysis of this subgroup, a multiple regression was fitted with MiCog total score as the dependent variable, PTA (bilateral average) as the primary predictor of interest, and SSQ12 total score, age, sex, and noise exposure as adjustment covariates (n = 60 complete cases). Because the MiCog total score is a bounded count variable (sum of 24 items, range 0–24) concentrated near zero, a negative binomial regression was fitted alongside the linear model as a sensitivity analysis. Neither PTA (B = 0.095, 95% CI −0.075 to 0.264, *p* = 0.266 in the linear model; IRR = 1.016, 95% CI 0.989–1.042, *p* = 0.247 in the negative binomial model) nor SSQ12, age, sex, or noise exposure were significantly associated with MiCog score in this adjusted model. The overall linear model did not reach statistical significance (R^2^ = 0.061; F (5.54) = 0.70; *p* = 0.623). No relevant collinearity was detected among predictors (VIF < 2 for all variables). Applying a Benjamini–Hochberg correction across the five predictors did not alter this conclusion (all adjusted *p* ≥ 0.83). Therefore, the adjusted analysis did not demonstrate an independent association between PTA and MiCog score.

Finally, among the 38 participants with both questionnaires, patient-reported MiCog and informant-reported SuCog scores were strongly correlated (r = 0.896, ρ = 0.879; both *p* < 0.001), and informants reported slightly more cognitive changes than patients (SuCog 10.4 ± 6.7 vs. MiCog 9.0 ± 6.7; paired t(37) = −2.87, *p* = 0.007; Wilcoxon *p* = 0.014). These findings support substantial convergent validity between self-reported and informant-reported cognitive complaints.

## 4. Discussion

This study provides an integrated characterization of auditory function in adults reporting subjective hearing complaints, combining objective audiometric measures, hearing-related quality of life, and, in a subgroup of older adults (≥60 years), cognitive screening. In relation to the first aim—to investigate the relationship between audiometric thresholds, SSQ12 scores, and cognitive screening outcomes—our findings indicate that these domains are interrelated but provide partially distinct information about auditory functioning. Consistent with the second aim—to assess the influence of age and environmental noise exposure on auditory performance—audiometric thresholds were consistently associated with age across the broad age range examined (18–90 years), with approximately 6 dB HL higher PTA4 per decade after adjustment for clinical covariates, supporting the well-established contribution of age-related hearing loss to auditory decline in adulthood [5,36,37,38,39]. Self-reported environmental noise exposure was associated with poorer SSQ12 scores, rather than with pure-tone thresholds, suggesting that noise exposure may relate more closely to perceived hearing difficulties in daily listening situations than to peripheral auditory sensitivity as measured by conventional audiometry [40]. Regarding the third aim—to identify potential indicators of early auditory dysfunction in individuals with normal conventional audiometry—participants with normal pure-tone thresholds nonetheless exhibited SSQ12 scores below the established threshold for normal self-reported hearing ability, suggesting the presence of subjective hearing difficulties not captured by conventional audiometry. This finding is consistent with previous evidence indicating that conventional audiometry may fail to capture early or more complex aspects of auditory dysfunction and highlights the value of self-reported measures such as the SSQ12 for detecting auditory difficulties in individuals with clinically normal hearing thresholds. Finally, in relation to the fourth aim, no significant association was found between hearing-related quality of life (SSQ12) and subjective cognitive complaints, and the unadjusted association between right-ear PTA and MiCog was ear-dependent and was not independent of SSQ12, age, sex and noise exposure in the adjusted model. Thus, the present data do not demonstrate an independent association between hearing measures and subjective cognitive complaints in older adults. All these results are discussed in detail below.

### 4.1. Functional Impact of Hearing Loss: SSQ12 Correlates and Predictive Value

In line with the first aim of this study, mean SSQ12 scores (5.64 ± 2.3) fell below the proposed clinical threshold for self-reported hearing disability. This threshold is consistent with cut-off values reported across independent validations of the scale—ranging from ≤6.0 for moderate-to-severe loss to ≤8.0–8.5 for any audiometrically confirmed impairment—which yield sensitivities of 78–80% and specificities of 76–80% for detecting elevated PTA [32,33,34]. This result was expected given that subjective hearing impairment was an inclusion criterion. SSQ12 scores were inversely associated with audiometric severity across the sample, supporting the instrument’s construct validity [32,34] and its value as a complementary measure to conventional audiometric testing.

SSQ12 correlated negatively with bilateral PTA (ρ = −0.532 to −0.484, *p* < 0.001) and age (ρ = −0.283, *p* < 0.001), consistent with the construct and discriminant validity demonstrated across the original and cross-linguistic validations of the scale, which reliably differentiate SSQ12 scores by hearing status [32,33,34] and with the moderate SSQ12–PTA correlations (r = −0.36 to −0.39) reported in normal-hearing adults by Kamerer et al. [41]. The age association may reflect combined peripheral and central decline [42]; the fact that the noise-exposure effect on SSQ12 scores remained significant after adjusting for audiometric severity, age, and sex suggests that self-reported noise exposure is associated with supra-threshold auditory deficits—difficulties not indexed by pure-tone audiometry—rather than simply reflecting pure-tone sensitivity. This conclusion is reinforced by the multiple regression reported in Section 3.4, in which noise exposure remained an independent predictor of lower SSQ12 scores (B = −1.005, *p* < 0.001) after adjusting for bilateral PTA, age, sex, and tinnitus, indicating that this association is not attributable to differences in audiometric severity, age, sex or tinnitus between exposed and non-exposed participants. We also note that female sex was independently associated with lower SSQ12 scores in this adjusted model; as this was not a pre-specified hypothesis, we report it here descriptively and flag it as a candidate for confirmation in future, adequately powered studies, rather than offering a substantive interpretation [20,43].

This mirrors findings in adults with audiometrically normal hearing, where a history of impulse-noise exposure—together with speech-intelligibility and temporal-processing measures—predicted SSQ12 scores, explaining up to 40% of their variance, and discriminating self-reported disability with 89% sensitivity and 86% specificity despite normal thresholds [41]. These data support the SSQ12’s clinical utility as a low-burden screening and monitoring tool that captures noise-related and supra-threshold auditory disability beyond what conventional audiometry detects, reinforcing its use alongside—rather than in place of—the audiogram [32,41]. Tinnitus presence was additionally tested as a covariate in the adjusted SSQ12 regression model (Section 3.4) and was not an independent predictor of SSQ12 scores (β = −0.008, 95% CI–0.554 –0.538; *p* = 0.978), while noise exposure, PTA4, and sex remained significant. In the literature, the tinnitus–SSQ12 association has been reported as borderline or inconsistent depending on sample characteristics [41]. In larger cohorts, tinnitus annoyance was found to be significantly associated with lower SSQ12 scores [44]. This suggests that sample size and tinnitus severity or annoyance, rather than its mere presence, may influence the strength and detectability of the tinnitus–SSQ12 association. Ototoxic exposure could not be evaluated as a covariate given its very small subgroup size (n = 4) in the main sample, which precludes any meaningful assessment of its association with SSQ12 scores.

### 4.2. Age and Audiometric Thresholds

Regarding the second aim of this study, the positive association between age and PTA4 in the ear-specific models with PTA4 as the outcome (approximately 0.6 dB HL per year in each ear) is consistent with aging as a major factor associated with auditory deterioration [3,5,38,39]. Given the cross-sectional design, this association should be interpreted as an association rather than as evidence of causation. Among the otologic covariates, otosclerosis and serous otitis media were associated with markedly higher PTA4 relative to normal otologic findings, as expected from their conductive component, whereas the association with age remained essentially unchanged after adjustment for these conditions. This pattern suggests that the observed age–PTA association predominantly reflects the sensorineural component of age-related hearing loss rather than being driven by superimposed conductive pathology. Other diagnoses, such as cholesteatomatous chronic otitis media or acoustic neuroma, were too infrequent to be evaluated, and larger conductive and mixed hearing-loss subgroups would be required to more confidently exclude residual confounding from these less prevalent etiologies.

However, presbycusis is not exclusively a peripheral process. Age-related changes in efferent inhibition, neural synchrony, and temporal processing—which were not assessed in the present study—may further contribute to speech-in-noise difficulties that are not fully captured by audiometric thresholds alone [42].

The near-perfect correlation between PTA4 and total PTA within each ear (right ear: r = 0.989; left ear: r = 0.987; both *p* < 0.001) indicates substantial redundancy between these measures. In contrast, the correlation between ears was moderate-to-strong rather than near-perfect (PTA4 ρ = 0.626; ρ = 0.54–0.76 across single frequencies). Although no systematic right–left difference was found at group level, about one quarter of participants had an interaural PTA4 difference of 15 dB or more, which is plausible in a clinical sample that includes unilateral middle-ear disease, otosclerosis and acoustic trauma. Findings obtained in one ear therefore cannot be assumed to represent the other ear, which is why results are reported separately for each ear.

In relation to the third aim—to identify potential indicators of early auditory dysfunction in individuals with normal conventional audiometry—participants with normative pure-tone thresholds nonetheless showed reduced SSQ12 scores. Because these participants were included specifically based on self-reported hearing difficulties, this finding is expected by design rather than constituting independent evidence of early or hidden auditory dysfunction. Establishing that the SSQ12 (or any other self-report measure) can detect dysfunction not captured by pure-tone audiometry (PTA) would require corroboration using objective suprathreshold measures (e.g., speech-in-noise testing, auditory processing measures, or electrophysiological indices), which were not available in the present study.

Nevertheless, this finding may have relevant clinical utility, particularly in otolaryngology services or clinical settings that do not have access to such resources. In these contexts, a reduced SSQ12 score may help identify patients with normal conventional audiometry who report hearing difficulties and who may therefore benefit from more detailed clinical assessment or closer follow-up. This potential application should be regarded as a supportive tool for patient identification and prioritization rather than as a diagnostic marker of early or hidden auditory dysfunction. This pattern is consistent with an expanding body of literature highlighting a disconnect between audiometric normality and everyday listening difficulty. Mealings et al. [45] reported that a substantial proportion of adults with normal or near-normal audiograms experience persistent difficulties understanding speech in noisy environments, yet are rarely offered a clinical management pathway, highlighting a clinical gap in current audiological practice. Similarly, Kamerer et al. [41] found that self-reported hearing disability, measured with the SSQ12, was not explained by pure-tone thresholds but was instead predicted by factors such as noise exposure history and suprathreshold measures, accounting for a substantial proportion of the variance in SSQ12 scores. Lunardelo et al. [46] similarly showed that individuals without audiometric hearing loss reported their greatest difficulties specifically in the noise-intelligibility domain of self-report questionnaires, even when speech-in-noise test performance did not fully account for these complaints. Finally, Mealings et al. [45] demonstrated that amplification could meaningfully reduce self-perceived listening difficulties in this population, suggesting that the difficulties captured by self-report tools such as the SSQ12 reflect a clinically meaningful and treatable form of auditory dysfunction. Taken together, these findings support the use of self-report measures such as the SSQ12 as complementary indicators of everyday listening difficulties that may warrant further assessment.

### 4.3. Noise Exposure Across Audiometric, Subjective, and Cognitive Measures

At the audiometric level, self-reported noise exposure was not significantly associated with PTA4 after adjustment for age and clinical covariates (right ear: B = 4.23, *p* = 0.069; left ear: B = 4.23, *p* = 0.063). This dissociation is consistent with the broader literature on the auditory impact of noise exposure: self-reported noise-exposure history is a relatively imprecise retrospective measure, while conventional PTA may fail to capture the full consequences of noise-related cochlear or supra-threshold dysfunction, particularly changes affecting the basal cochlear region or frequencies outside the conventional audiometric range [46,47,48]. The absence of an independent association between noise exposure and PTA should therefore not be interpreted as evidence of an absence of auditory effects.

By contrast, at the subjective level, greater noise exposure was independently associated with lower SSQ12 scores after adjusting for audiometric severity, age, sex, and tinnitus (B = −1.005, 95% CI −1.565 to −0.446; *p* < 0.001; Section 3.4), indicating that this association is not attributable to differences in hearing-threshold severity between exposed and non-exposed participants. This SSQ12 association indicates that self-reported measures capture aspects of everyday hearing difficulty not reflected in pure-tone thresholds and is consistent with previous studies showing that noise-exposure history predicts self-reported disability independently of preserved PTA [41]. These findings are relevant to the third aim of identifying indicators of auditory dysfunction not captured by normal conventional audiometry and support the complementary use of self-reported measures alongside conventional audiometry when evaluating the auditory consequences of noise exposure.

At the cognitive level, however, MiCog scores did not differ significantly between participants with and without a history of noise exposure when restricted to the n = 60 cognitive subgroup (t(58) = −0.288, *p* = 0.774; Mann–Whitney U = 417, *p* = 0.988), indicating no measurable association between self-reported noise exposure and cognitive screening outcomes in this sample. This null finding contrasts with the wider literature, in which chronic noise exposure has been associated with cognitive decline through mechanisms that plausibly overlap with those relating hearing loss to cognition [49]. A recent systematic review and meta-analysis of longitudinal cohorts reported a 15% increase in dementia risk per 10 dB increment in environmental noise exposure (HR = 1.15, 95% CI 1.03–1.28) [50], and population-based data from the China Health and Retirement Longitudinal Study similarly associated higher noise exposure to lower global cognitive scores, with an effect magnitude comparable to that of hypertension or several years of aging [48]. However, both reviews emphasize substantial heterogeneity across studies in exposure metrics, cognitive outcome measures, and confounder adjustment, precluding firm causal conclusions. Our null result may therefore reflect the imprecision of retrospective self-reported noise exposure, which is subject to recall bias even when moderate agreement with objective exposure metrics has been reported [51]; the restricted size of the cognitively characterized subgroup (n = 60); or a genuine absence of association within this sample—consistent with reports that the association between environmental noise exposure and cognitive function remains variable across the literature [50]. This question should be revisited in future studies using more precise cumulative noise-exposure metrics and larger cognitively characterized cohorts.

Overall, these results indicate that the auditory consequences of noise exposure are more readily captured by subjective reports than by conventional thresholds, whereas no measurable cognitive correlation emerged in this sample—reinforcing the value of an integrated assessment combining audiometry with self-reported measures in noise-exposed populations.

### 4.4. Hearing Loss and Cognitive Screening

Regarding the fourth aim of this study—exploring the relationship between hearing-related quality of life (HRQoL) and subjective cognitive complaints—no significant association was found between the SSQ12 total score and MiCog in the older-adult subgroup (ρ = −0.241, *p* = 0.064), and SSQ12 was not a significant predictor in the adjusted model. An exploratory item-level analysis likewise showed no individual SSQ12 item significantly associated with MiCog, although this analysis is limited by the unfavorable cases-to-predictors ratio of a 12-predictor model fitted on 60 observations. Based on the present data, therefore, neither global nor item-level self-reported hearing difficulty reliably tracked subjective cognitive complaints.

With respect to audiometric thresholds, right-ear PTA showed an unadjusted correlation with MiCog (ρ = 0.332, *p* = 0.010), directionally consistent with the auditory–cognitive literature, which reports small associations between hearing loss and both objective and self-reported cognitive performance in aging populations [52,53]. However, this association was not observed for the left ear (ρ = 0.109, *p* = 0.409) or the better ear (ρ = 0.207, *p* = 0.112), and it did not persist in the principal multivariable regression adjusting for SSQ12 score, age, sex, and noise exposure (linear model: B = 0.095, 95% CI −0.075 to 0.264, *p* = 0.266; negative binomial model: IRR = 1.016, *p* = 0.247). None of the other predictors was significant, and the overall model did not reach significance (R^2^ = 0.061, *p* = 0.623). Therefore, the adjusted analysis did not demonstrate an independent association between audiometric thresholds and subjective cognitive complaints. The limited size of the cognitive subgroup may have contributed to this result: with n = 60 and five predictors, the model had 80% power to detect only moderate effects (partial r ≈ 0.35), whereas the partial correlation observed for PTA was small (partial r ≈ 0.15). Smaller effects, such as those typically reported for hearing–cognition associations, therefore cannot be excluded, and larger studies are needed to address this question.

In an exploratory analysis, the right-ear association appeared stronger at 250 Hz–2 kHz (r = 0.26–0.32, *p* ≤ 0.046) than at 4–8 kHz (r ≤ 0.08, *p* ≥ 0.56). The direct comparison of these correlations reached nominal significance (Steiger’s Z = 2.02, *p* = 0.043; bootstrap 95% CI for the difference 0.01–0.55; Section 3.5). However, given the approximate nature of the test for Spearman coefficients, the absence of multiplicity correction, the ear dependence described above and the modest subgroup size, this pattern should be regarded only as a hypothesis for future studies. If confirmed, it would be compatible with the mechanisms proposed to link hearing loss and cognition, which include shared age-related neurodegeneration, sensory deprivation of central auditory pathways, and increased cognitive load [52,53]. In particular, it would fit the effortful listening hypothesis—formalized in the Framework for Understanding Effortful Listening (FUEL) and the Ease of Language Understanding (ELU) model—whereby degraded speech perception, which depends on low-to-mid-frequency audibility, may consume working-memory and executive resources that would otherwise support encoding [54,55].

These findings should also be interpreted within the inherent limitations of subjective cognitive screening instruments. The SCD-Q, comprising the self-reported MyCog (MiCog) and informant-reported TheirCog (SuCog) forms, was developed and validated by Rami et al. as a measure of subjective cognitive decline rather than as a stand-alone diagnostic test of cognitive impairment. In the original validation study, MyCog discriminated participants with subjective cognitive decline from controls, whereas TheirCog showed stronger associations with objective neuropsychological performance and, at a cutoff of 7/24, demonstrated 85% sensitivity and 80% specificity for discriminating individuals with and without cognitive impairment. Importantly, MyCog scores were not significantly correlated with neuropsychological test performance in that study [22]. In our sample, self- and informant-reported scores were strongly correlated (r = 0.896), although informants reported slightly more cognitive changes than patients (Section 3.5). Accordingly, neither the MiCog total score nor its association with audiometric or SSQ12 measures should be interpreted as evidence of cognitive impairment; elevated scores should instead be regarded as indicators of subjective cognitive complaints that may warrant further clinical or neuropsychological assessment.

Although no independent association between hearing measures and subjective cognitive complaints was demonstrated, brief cognitive screening within audiology may still have practical value: it can flag patients who may need slower-paced counseling, simplified instructions, or closer follow-up during hearing aid fitting or rehabilitation programs, and can help clinicians distinguish auditory from cognitive contributions to reported communication difficulty rather than attributing all complaints to threshold loss [10,56]. Whether auditory rehabilitation influences cognitive outcomes cannot be addressed with the present cross-sectional data.

### 4.5. Study Strengths and Limitations

The relatively large, well-characterized sample spanning a wide age range (18–90 years), complemented by a focused subgroup analysis of older adults incorporating cognitive screening, strengthens the generalizability of the findings across the spectrum of auditory aging. The multidimensional protocol—audiometry, validated SSQ12, and dual patient/informant cognitive screening—exceeds audiometry-only characterization, with informant corroboration mitigating self-report limitations. Frequency-specific and ear-specific analyses allowed us to generate an exploratory hypothesis regarding speech-relevant frequencies and to show that some associations (notably PTA–MiCog) were ear-dependent, which prevents over-interpretation of single-ear findings. Using simultaneous-entry regression and collinearity diagnostics to interrogate stepwise-derived findings guards against overinterpreting item-level artifacts.

On the other hand, the main limitations of this work include the following: the cognitive subgroup’s modest size (n = 60) limited statistical power for the exploratory item and frequency-level analyses; in the principal adjusted regression, no independent association between PTA and MiCog scores was demonstrated, although small effects cannot be excluded; the very small ototoxic-exposure subgroup in the main sample (n = 4) similarly precluded a meaningful evaluation of this variable in relation to SSQ12 scores. The cross-sectional design precludes causal inference, and consecutive clinical sampling from a single center may limit generalizability. Missing data across the PTA-related regression models were handled with a complete-case (listwise deletion) approach rather than imputation; given the low overall proportion of missing values (2.2–3.4% across these models), we considered this preferable to imputation methods that could introduce additional uncertainty. The absence of validated speech-in-noise testing precluded correlating self-report and cognitive measures with objective suprathreshold processing [57]. Finally, MiCog is a screener, not a diagnostic tool, and its scores indicate cognitive risk rather than a defined impairment type or severity [22]. Potentially relevant factors that may influence subjective cognitive complaints—including educational attainment, socioeconomic status, depressive or anxiety symptoms, vascular comorbidities, and medication use—were not collected and could not be adjusted for in these analyses; their absence should be considered when interpreting the MiCog and SuCog findings.

Finally, availability of a completed MiCog assessment within the ≥60-year stratum was itself non-random: participants with a completed MiCog were significantly older (72.3 ± 8.1 vs. 62.6 ± 2.2 years, *p* < 0.001) and had significantly poorer right- and left-ear PTA4 (*p* < 0.001 and *p* = 0.019, respectively) than those without, although sex, noise-exposure history, and SSQ12 scores did not differ (Table 2). This pattern is a potential source of selection bias in the cognitive-subgroup analyses and should be borne in mind when generalizing these findings beyond patients with more severe, longer-standing hearing loss.

Future research could benefit from larger, multicenter datasets and more robust statistical/analytical approaches. With this aim, open and collaborative datasets may improve the ability to better characterize patients with atypical or individual clinical profiles. A structured dataset containing all the study variables used in our study, comprising a sample of 178 subjects and about 30 variables related to audiological, quality of life and cognitive characteristics, is openly available in Zenodo at https://doi.org/10.5281/zenodo.23281821.

## 5. Conclusions

This study provides an integrated characterization of auditory function by jointly considering audiometric thresholds, self-reported hearing-related quality of life, and cognitive screening outcomes. The findings demonstrate that these domains are related but only partially overlapping, with each contributing distinct information to the overall auditory profile. SSQ12 scores were significantly associated with bilateral PTA (ρ = −0.532 to −0.484, *p* < 0.001), supporting their construct validity and clinical utility as a complement to, rather than a substitute for, conventional audiometry.

Age was consistently associated with higher audiometric thresholds (approximately 0.6 dB HL per year in each ear; right ear: B = 0.599, 95% CI 0.469–0.729; left ear: B = 0.609, 95% CI 0.483–0.735; both *p* < 0.001), whereas environmental noise exposure was not independently associated with PTA. Instead, noise exposure showed a consistent association with self-reported SSQ12 hearing difficulty that remained significant after adjusting for PTA, age, sex, and tinnitus (B = −1.005, *p* < 0.001), thus suggesting that age and noise exposure may be related to auditory function through distinct pathways that are not equally captured by conventional audiometry. This independent association between noise exposure and lower SSQ12 scores suggests that conventional audiometry does not fully capture the burden of auditory dysfunction.

Finally, in the cognitive subgroup, MiCog showed an unadjusted correlation with right-ear PTA (ρ = 0.332, *p* = 0.010) that was not observed for the left ear and did not persist after adjustment for SSQ12, age, sex and noise exposure (B = 0.095, *p* = 0.266). Thus, the adjusted analysis did not demonstrate an independent association between audiometric thresholds and subjective cognitive complaints. The apparent predominance of low-to-mid frequencies in the right-ear association was exploratory and requires confirmation. No significant association was found between MiCog and the total SSQ12 score, either as a zero-order correlation (ρ = −0.241, *p* = 0.064) or in the adjusted regression.

Taken together, these findings indicate that no single measure—audiometric, self-reported, or cognitive—fully captures the burden of auditory dysfunction on its own; each offers a complementary lens on a patient’s hearing-related difficulties. Incorporating brief self-reported and cognitive screening tools alongside conventional audiometry may therefore support earlier identification of patients who would benefit from closer follow-up or further evaluation, without replacing either domain diagnostically. This study provides open access to a dataset with more than 30 variables collected in 178 subjects, hopefully promoting further use and research.

## Figures and Tables

**Figure 1:**
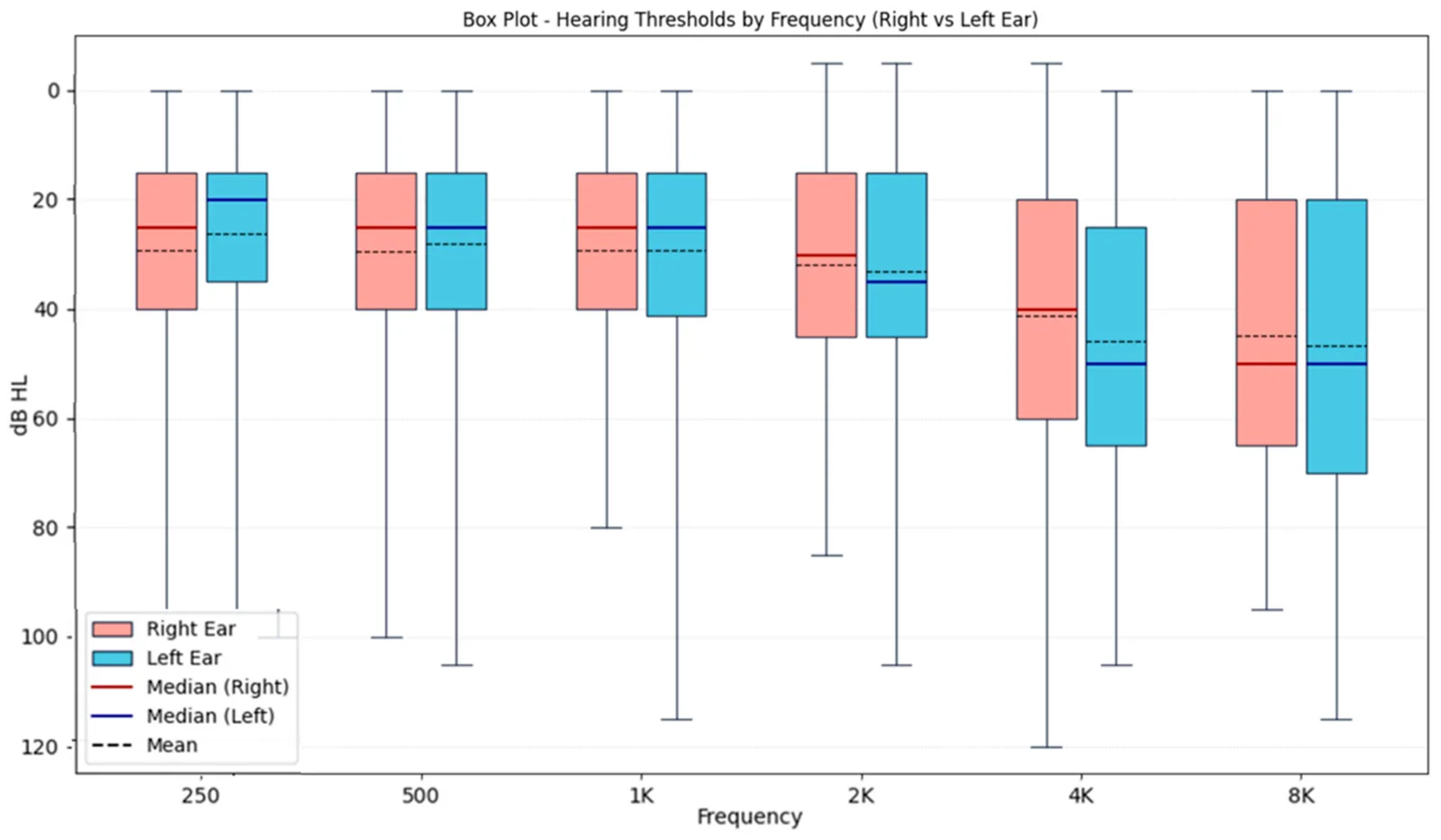
Distribution of hearing thresholds (dB HL) by frequency for the right and left ears. Boxes represent the interquartile range (P25–P75), the solid line indicates the median (P50), and the dashed line indicates the mean. Whiskers extend to the minimum and maximum values. The y-axis is shown inverted to follow standard audiometric convention, with lower thresholds (better hearing) plotted higher on the axis.

**Figure 2:**
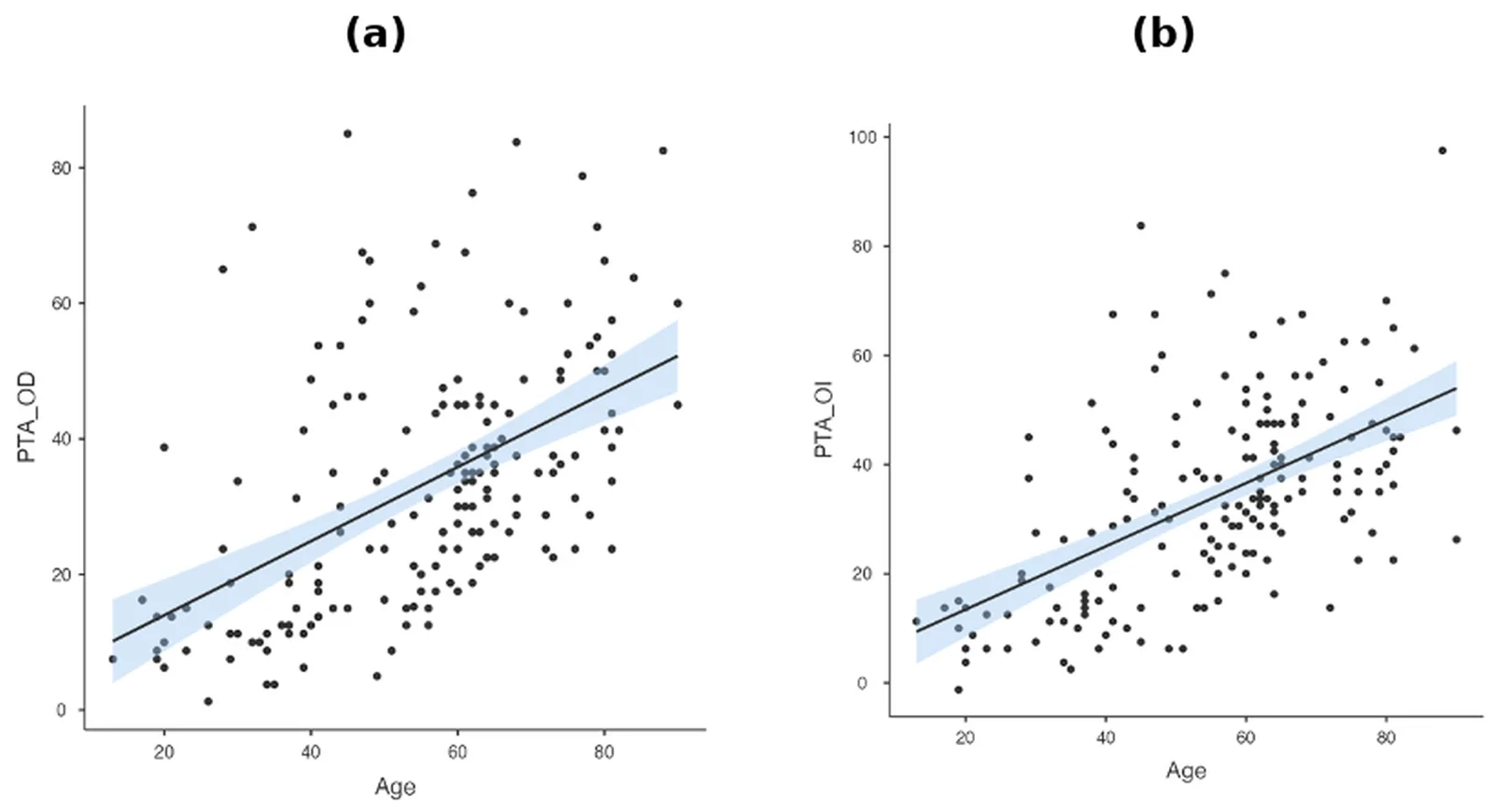
Scatter plots showing the association between age and pure-tone average (PTA) hearing thresholds for the right ear (PTA_OD, panel (**a**)) and left ear (PTA_OI, panel (**b**)). Each point represents an individual participant. The solid line represents the fitted linear regression, and the shaded area denotes the 95% confidence interval. Both panels show a positive association between age and PTA, indicating poorer hearing thresholds with increasing age in both ears (PTA_OD: Pearson’s r = 0.515, *p* < 0.001; PTA_OI: Pearson’s r = 0.562, *p* < 0.001).

**Figure 3:**
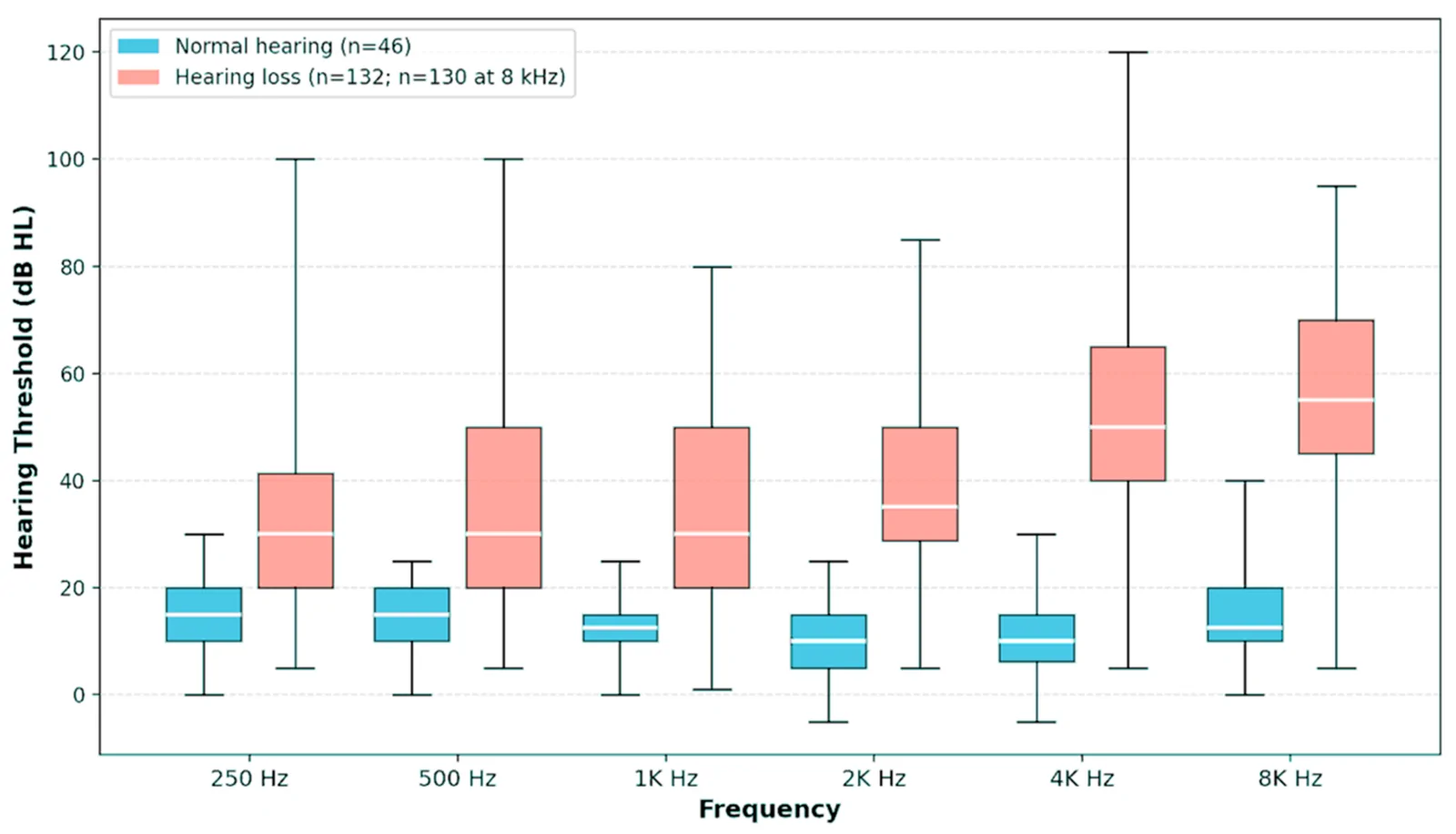
Distribution of audiometric thresholds (dB HL) by frequency in the right ear, stratified by hearing status.

**Figure 4:**
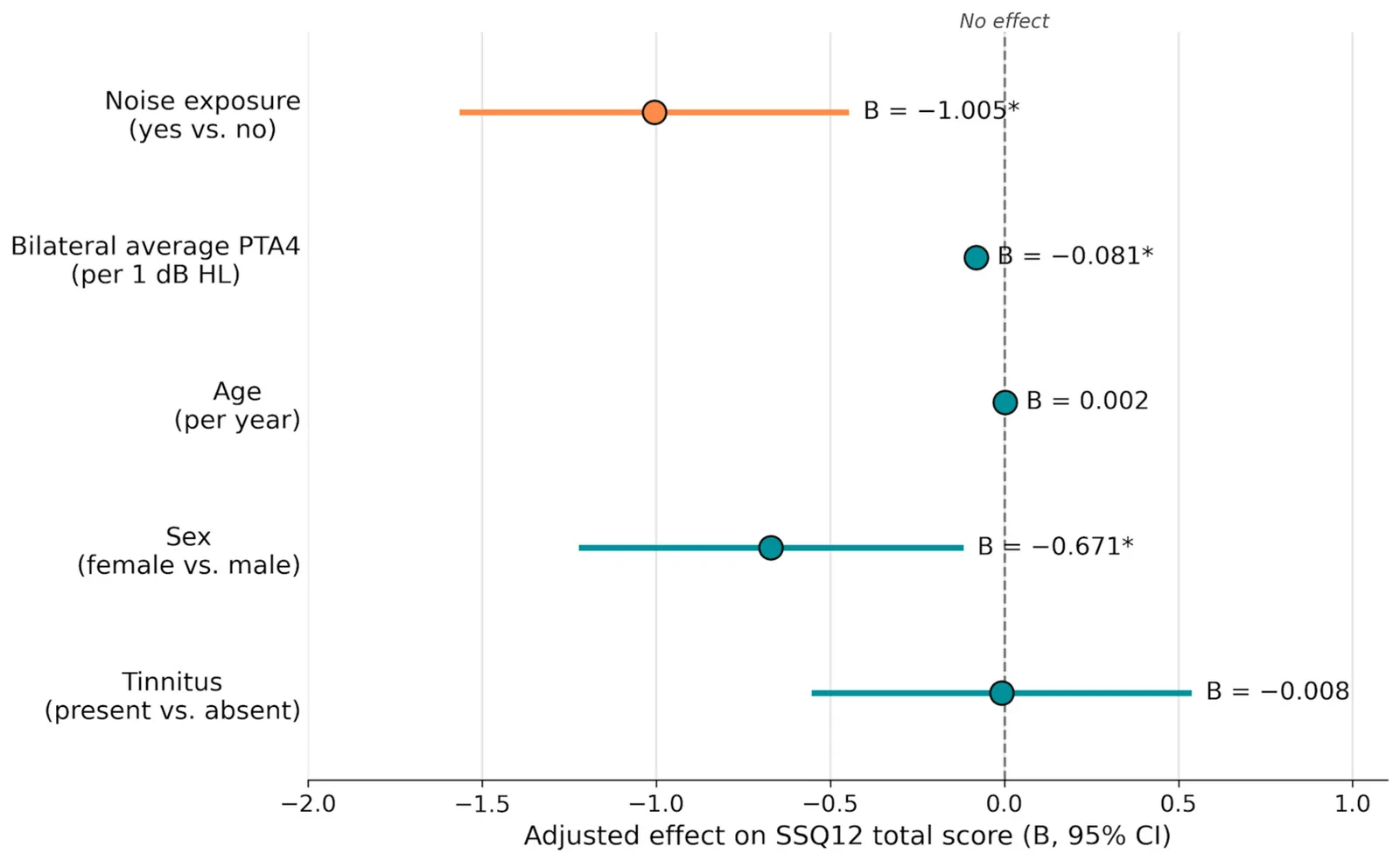
Adjusted associations with SSQ12 total score from the multivariable regression model (N = 177, R^2^ = 0.394). Points show regression coefficients (B) with 95% confidence intervals; the dashed line marks no effect. * *p* < 0.05.

**Figure 5:**
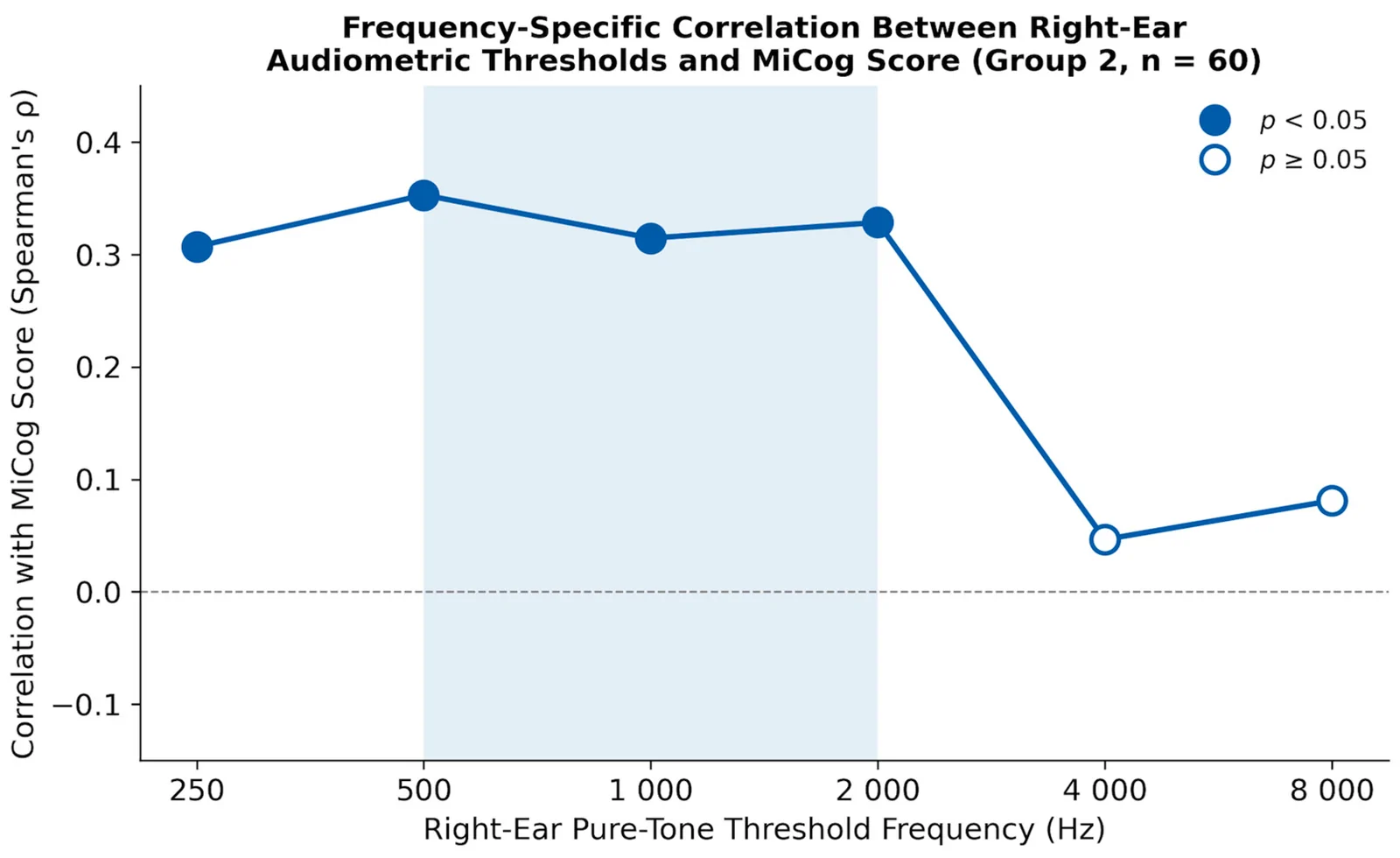
Frequency-specific correlation between right-ear pure-tone thresholds and MiCog cognitive screening score (Subgroup, n = 60). Filled circles indicate statistically significant correlations (*p* < 0.05); open circles indicate non-significant correlations. The shaded region (500 Hz–2 kHz) highlights the frequency range driving the association, contrasting with the non-significant high-frequency thresholds (4–8 kHz).

**Table 1:** Demographic and clinical characteristics of participants.

Variable	Value	N
Demographic variables		
Age, years, mean ± SD (range)	55 ± 17.4 (18–90)	177
Sex, male, n (%)	77 (43.3%)	178
Sex, female, n (%)	101 (56.7%)	178
Clinical history		
Environmental noise exposure, n (%)	70 (39.3%)	178
Ototoxic agent exposure, n (%)	4 (2.2%)	178
Vestibular disorders/symptoms, n (%)	25 (14.1%)	178
Use of hearing assistive devices, n (%)	2 (1.1%)	
Oto-audiological clinical variables		
Otologic findings normal, right ear, n (%)	161 (90.4%)	178
Otologic findings normal, left ear, n (%)	158 (88.7%)	178
Chronic middle ear disease, right ear, n (%)	22 (12.3%)	178
Chronic middle ear disease, left ear, n (%)	26 (14.6%)	178
History of otologic surgery, right ear, n (%)	1 (0.6%)	178
History of otologic surgery, left ear, n (%)	5 (2.8%)	178
Hearing loss, right ear, n (%)—of which conductive/mixed	132 (74.2%)—8 (6.0%)/13 (9.8%)	178
Hearing loss, left ear, n (%)—of which conductive/mixed/cophosis	136 (76.4%)—10 (7.3%)/15 (11.0%)/2 (1.5%)	178
Tinnitus, n (%)	88 (49.4%)	178
PTA4, right ear, dB HL, mean ± SD (range)	33.0 ± 18.6 (1.3–85.0)	178
Total PTA, right ear, dB HL, mean ± SD (range)	33.9 ± 17.9 (3.3–86.7)	176
PTA4, left ear, dB HL, mean ± SD (range)	33.7 ± 18.0 (−1.25–97.5)	175
Total PTA, left ear, dB HL, mean ± SD (range)	34.4 ± 17.9 (0.83–99.2)	174
Air-bone gap, right ear (conductive losses), dB, mean ± SD (range)	24.2 ± 8.4 (15–45)	19
Air-bone gap, left ear (conductive losses), dB, mean ± SD (range)	26.1 ± 9.3 (15–45)	23
Self-administered questionnaire		
SSQ12 total score, mean ± SD (range)	5.64 ± 2.3 (0.8–10)	178
MiCog score, mean ± SD (range)	7.9± 6.6 (range 0–24)	60
SuCog score, mean ± SD (range)	10.4 ± 5.3 (range 0–24)	38

**Table 2:** Comparison of demographic and clinical characteristics according to the availability of MiCog assessment among participants aged ≥ 60.

Variable	“MiCog” Documented (n = 60)	“MiCog” Not Documented (n = 25)	*p*-Value
Demographic variables			
AgeOk	72.3 ± 8.14 (60–90)	62.6 ± 2.18 (60–68)	<0.001
Gender	30 (50%)/30 (50%)	12 (48%)/13 (52%)	0.867
Clinical History			
Environmental exposure to intense noise	22 (36.7%)	8 (32%)	0.966
Auditory disorders right ear	60 (100%)	24 (96%)	0.294
Auditory disorders left ear	60 (100%)	25 (100%)	—
Vestibular disorders	12 (20%)	2 (8%)	0.216
Use of hearing assistive devices			
Oto/Audiological Clinical Variables			
PTA right ear	44.3 ± 15.8 (18.8–83.8)	32.5 ± 7.5 (17.5–45)	<0.001
PTA left ear	43.9 ± 13.8 (13.8–97.5)	36.8 ± 12.7 (16.3–66.3)	0.019
Presence of tinnitus	30 (50%)	12 (48%)	0.867
Self-administered Questionnaires			
Total SSQ12 score	4.9 ± 2.3 (1.3–9.8)	5.6 ± 1.7 (3.6–8.3)	0.183

**Table 3:** Adjusted associations with SSQ12 total score from the multivariable regression model shown in Figure 4 (N = 177, R^2^ = 0.394, adjusted R^2^ = 0.377). B, unstandardized regression coefficient; SE, standard error of β; CI, confidence interval.

Predictor	B	SE	95% CI	*p*-Value
Noise exposure (yes/no)	−1.005	0.283	[−1.565, −0.446]	<0.001
Bilateral average (per 1 dB HL)	−0.081	0.010	[−0.101, −0.061]	<0.001
Age (per year)	0.002	0.010	[−0.017, 0.022]	0.822
Gender (female/male)	−0.671	0.280	[−1.223, −0.118]	0.018
Tinnitus (yes/no)	−0.008	0.277	[−0.554, 0.538]	0.978

## Data Availability

The anonymized participant-level dataset supporting the findings of this study, comprising 178 subjects and more than 30 study variables, is openly available in Zenodo at https://doi.org/10.5281/zenodo.23281821.

## Supplementary Materials

The following supporting information can be downloaded at: https://www.mdpi.com/article/10.3390/audiolres16050150/s1, Table S1: Study variables; Table S2: Statistical analyses performed, classified as primary or exploratory; Table S3: Effect sizes, 95% confidence intervals, and raw and Benjamini–Hochberg-adjusted *p*-values for the primary, aim-linked analyses.

## Abbreviations

The following abbreviations are used in this manuscript:

ABG	Air-Bone GAP
AC	Air-conduction
B	Unstandardized regression coefficient
BC	Bone-conduction
AIC	Akaike Information Criterion
CI	Confidence Interval
dB	Decibel
ELU	Ease of Language Understanding
FUEL	Framework for Understanding Effortful Listening
GDPR	General Data Protection Regulation
HC3	Heteroscedasticity-Consistent standard errors (type 3)
HL	Hearing Level
HRQoL	Hearing-Related Quality of Life
IEC	International Electronical Commission
IRR	Incidence Rate Ratio
ISO	International Organization for Standardization
MiCog	Self-report from of the SCD-Q (“Mi cognición”)
PTA	Pure-Tone Average
SCD-Q	Subjective Cognitive Decline Questionnaire
SD	Standard Deviation
SE	Standard Error
SSQ12	Speech, Spatial and Qualities of Hearing Scale (12)
SuCog	Informant-report form of the SCD-Q (“Su cognición”)
VIF	Variance Inflation Factor
WHO	World Health Organization
