# Supplementary material for: Audiometric, Perceptual and Subjective Cognitive Assessment of Hearing Loss in Adults with Hearing-Related Complaints
Source: Audiol Res. 2026 Oct 1;16(5):150. doi: 10.3390/audiolres16050150 (PMC13701541; doi:10.3390/audiolres16050150)
Supplement: Supplementary file 1 [file audiolres-16-00150-s001.zip › audiolres-4550435-supplementary.pdf]

<u>Supplementary material</u>

Table S1. Study variables.

Variable	Type of variable	Values (units)
**Demographic variables**		
Age	Numerical	
Gender	Categorical	Male/Female
**Clinical History**		
Exposure to ototoxic agents	Categorical	Yes/No
Environmental exposure to intense noise	Categorical	Yes/No
Neurological disorders diagnoses	Categorical	Yes/No
Mental health diagnoses	Categorical	Yes/No
Otologic diagnoses	Categorical	Normal/simple chronic otitis media/cholesteatomatous chronic otitis media/serous otitis media/otosclerosis
Auditory disorders	Categorical	Yes/No
Vestibular disorders	Categorical	Yes/No
Use of hearing assistive devices	Categorical	Yes/No
**Oto/Audiological Clinical Variables**		
Hearing loss type	Nominal	Sensorineural, conductive or mixed
Presence of tinnitus	Categorical	Yes/No
Audiometric thresholds in both ears at frequencies 250, 500, 1000, 2000, 4000 and 8000 Hz	Numerical	dB HL
Mean audiometric air-bone gap	Numerical	dB HL
**Self-administered Questionnaires**		
Total SSQ12 score	Numerical	0–10
Individual SSQ12 items scores	Numerical	0–10
Global MiCog questionnaire score	Numerical	0–24
Individual MiCog items score	Numerical	0–1
Global SuCog questionnaire score	Numerical	0–24
Individual SuCog items score	Numerical	0–1

Table S2. Statistical analyses performed, classified as primary or exploratory.

Category	Test	Analyses performed	
Descriptive statistics	Descriptives	• Absolute frequencies and percentages<br>• Mean and standard deviation (SD)<br>• Median and range<br>• 95% confidence intervals	Descriptive
Normality	Shapiro-Wilk	• Distribution of continuous variables<br>• Normality of regression-model residuals	Descriptive
Correlations	Pearson	• PTA4 vs. total PTA (right and left ear)<br>• Correlations between adjacent audiometric frequencies (250–8000 Hz)<br>• PTA by frequency (250 Hz–2 kHz and 4–8 kHz) vs. MiCog<br>• MiCog vs. SuCog	Exploratory
	Spearman	• SSQ12 vs. right-ear PTA4 (N = 178)<br>• SSQ12 vs. left-ear PTA4 (N = 175)	**Primary**
	Spearman	• PTA4 vs. total PTA (right and left ear)<br>• Age vs. audiometric thresholds (250–8 kHz)<br>• Age vs. SSQ12<br>• PTA vs. MiCog	Exploratory
	Spearman (ear-based sensitivity analysis; subgroup n = 60)	• MiCog vs. PTA4 (bilateral average)<br>• MiCog vs. PTA4 (worse ear)<br>• MiCog vs. PTA4 (better ear)	Exploratory
	Zero-order correlation (Pearson / Spearman; subgroup n = 60)	• MiCog vs. right-ear PTA4<br>• MiCog vs. age<br>• MiCog vs. SSQ12 (total score)	**Primary**
	Comparison of dependent correlations (Steiger's Z)	MiCog–PTA correlation at low/mid frequencies (250 Hz–2 kHz) vs. high frequencies (4–8 kHz)	Exploratory
Independent groups	Student's t-test	SSQ12, normal hearing vs. hearing loss (N = 178)	**Primary**
	Student's t-test	• Right-ear PTA4, left-ear PTA4, right-ear total PTA and left-ear total PTA, by noise exposure<br>• Age by noise exposure<br>• MiCog by noise exposure (subgroup n = 60)	Exploratory
	Mann-Whitney U	SSQ12 by noise exposure, overall (N = 178)	**Primary**
	Mann-Whitney U	MiCog by noise exposure (subgroup n = 60; non-parametric check)	Exploratory
	Chi-square (χ^2)	Distribution of audiological diagnosis by noise exposure	Exploratory
Regression models	Multiple linear regression	Dependent: age. Main predictors: right- and left-ear PTA (N = 174). Covariates: noise exposure, tinnitus, right-ear otologic diagnosis, left-ear otologic diagnosis, and vestibular pathology	**Primary**

**Binary logistic regression**	Dependent: audiological diagnosis (normal hearing vs. hearing loss; N = 176). Predictors: hearing thresholds (250 Hz–8 kHz, incl. 4 kHz) and noise exposure	**Primary**
**Multiple linear regression**	Dependent: SSQ12 (total score). Main predictor: noise exposure. Covariates: bilateral PTA4, age, sex, and tinnitus	**Primary**
**Multiple linear regression + negative binomial regression (sensitivity analysis)**	Dependent: MiCog (total score). Main predictor: bilateral PTA (average). Covariates: SSQ12, age, sex, and noise exposure	**Primary**

Table S3. Effect sizes, 95% confidence intervals, and raw and Benjamini–Hochberg-adjusted p-values for the primary, aim-linked analyses.

Primary analysis (N)	Effect size [95% CI]	Raw p	BH-adjusted p
SSQ12 vs. right PTA4 (Spearman, N = 178)	ϱ = −0.532 [−0.630, −0.418]	< 0.001	< 0.001
SSQ12 vs. left PTA4 (Spearman, N = 175)	ϱ = −0.484 [−0.590, −0.362]	< 0.001	< 0.001
SSQ12, normal hearing vs. hearing loss (t-test, N = 178)	d = 0.989 [0.638, 1.340]	< 0.001	< 0.001
SSQ12 by noise exposure, overall (Mann–Whitney, N = 178)	r = 0.293	< 0.001	0.002
Age → PTA regression, right ear (N = 174)	β = 0.313 [0.152, 0.474]	< 0.001	< 0.001
Age → PTA regression, left ear (N = 174)	β = 0.371 [0.207, 0.536]	< 0.001	< 0.001
SSQ12 ~ noise, adjusted for PTA/age/sex/tinnitus (N = 177; Comment 11)	β = −1.005 [−1.565, −0.446]	0.001	0.001
4-kHz threshold, logistic regression (N = 176; Comment 12)	OR = 1.17 [1.05, 1.31]	0.005	0.006
MiCog vs. right PTA4 (Spearman, N = 60)	ϱ = 0.332 [0.085, 0.540]	0.010	0.012
MiCog vs. age (Spearman, N = 60)	ϱ = 0.044 [−0.212, 0.295]	0.736	0.736
MiCog vs. SSQ12 total (Spearman, N = 60)	ϱ = −0.241	0.064	0.070
